# Emotional Activity Is Negatively Associated With Cognitive Load in Multimedia Learning: A Case Study With EEG Signals

**DOI:** 10.3389/fpsyg.2022.889427

**Published:** 2022-06-13

**Authors:** Xiang Guo, Tianshui Zhu, Chennan Wu, Zongliang Bao, Yang Liu

**Affiliations:** School of Information and Electronic Engineering, Zhejiang University of Science and Technology, Hangzhou, China

**Keywords:** emotional activity, cognitive load, multimedia learning, emotion dynamics, EEG signals

## Abstract

We aimed to investigate the relationship between emotional activity and cognitive load during multimedia learning from an emotion dynamics perspective using electroencephalography (EEG) signals. Using a between-subjects design, 42 university students were randomly assigned to two video lecture conditions (color-coded vs. grayscale). While the participants watched the assigned video, their EEG signals were recorded. After processing the EEG signals, we employed the correlation-based feature selector (CFS) method to identify emotion-related subject-independent features. We then put these features into the Isomap model to obtain a one-dimensional trajectory of emotional changes. Next, we used the zero-crossing rate (ZCR) as the quantitative characterization of emotional changes *ZCR*_*EC*_. Meanwhile, we extracted cognitive load-related features to analyze the degree of cognitive load (CLI). We employed a linear regression fitting method to study the relationship between *ZCR*_*EC*_ and CLI. We conducted this study from two perspectives. One is the frequency domain method (wavelet feature), and the other is the non-linear dynamic method (entropy features). The results indicate that emotional activity is negatively associated with cognitive load. These findings have practical implications for designing video lectures for multimedia learning. Learning material should reduce learners’ cognitive load to keep their emotional experience at optimal levels to enhance learning.

## Introduction

Cognitive load theory (CLT) has been widely used in learning research to explain the learning process (Sweller, 1988, 1994). Cognitive load represents the load that performing a particular task imposes on a learner’s cognitive system (Paas et al., 1994). CLT is based on the cognitive architecture associated with working memory. Owing to the limitations of human working memory, inappropriate learning materials may cause high cognitive load (Paas and Sweller, 2014). Mayer introduced CLT to multimedia learning and established the cognitive theory of multimedia learning (CTML). However, this theory only focuses on the cognitive process and neglects the influence of emotion on learning.

Moreno extended the CTML by adding motivational and affective aspects and established the cognitive affective theory of learning with media (CATLM) (Moreno, 2007). The central theme in the CATLM is that in multimedia learning, learners’ cognitive processing is jointly affected by emotional, motivational, and metacognitive factors (Mayer and Estrella, 2014). Learning includes both affective and cognitive processes. Every information-processing step in the learning process is emotional and cognitive. Therefore, researchers began to study how affective and cognitive aspects may affect each other during multimedia learning.

Plass and Kaplan (2016) further proposed the Integrated Cognitive Affective Model of Learning (ICALM) based on the CATLM (Moreno and Mayer, 2007). The main hypothesis of this model is that affective and cognitive processes are intertwined and inseparable. The cognitive affective processing of multimedia learning materials demands cognitive resources. According to the unlimited capacity assumption, humans can handle an unlimited amount of material (Baddeley, 1992). Does an increase in cognitive load reduce emotional activity? So far as we know, this issue has not been studied from a neuroscientific perspective. In this study, we designed an experiment to investigate the relationship between cognitive load and emotional changes using EEG signals.

Emotion and cognitive load can be measured using subjective (rating scales) and objective techniques (physiological parameters). A survey found that the most common methods used to assess cognitive load are subjective measures (Duygu et al., 2019). Subjective measures include indirect types such as self-reported mental effort (Paas et al., 1994) and direct types such as material difficulty ratings (Kalyuga et al., 1999). The Achievement Emotions Questionnaire, Self-Assessment Manikin, and Positive and Negative Affect Schedule are the most popular questionnaires used in emotion studies (Elaheh et al., 2019). Subjective measures, however, have some limitations, such as limited reliability and validity (Brunken et al., 2003; Anmarkrud et al., 2019). For example, participants may not answer exactly in accordance to how they are feeling, but how they feel others would answer (Bethel et al., 2007).

Furthermore, theories on emotion dynamics suggest that the fundamental feature of emotions is that they change over time (Kuppens et al., 2010). Thus, by subjective measurement, people are characterized only in terms of how they feel, on average. Using physiological signals, such as electroencephalography (EEG), which are objective data, we can instantaneously and continuously measure emotional states. Thus, we can record how people’s emotions change over time. By doing this, we can better understand the participants’ underlying responses during the learning process.

Electroencephalography is a neuroimaging technique that can non-invasively measure brain activity in a real-world environment using electrodes placed on the scalp. Compared to other external appearance clues, such as gestures and facial expressions, EEG is a technology that is more reliable at recognizing emotion due to its higher accuracy and objective evaluation (Ahern and Schwartz, 1985). Various psychophysiological studies have demonstrated correlations between emotions and EEG signals (Sammler et al., 2007; Mathersul et al., 2008; Knyazev et al., 2010). Additionally, with the rapid development of wearable devices and dry electrode techniques (Chi et al., 2011; Grozea et al., 2011; Wang et al., 2012; Huang et al., 2015), EEG-based emotion recognition has the potential to be implemented in practical settings, such as mental state monitoring. In addition, EEG signals have been shown to provide informative signal features in response to emotional states (Harmony et al., 1996; Hammond, 2007; Heinrich et al., 2007). Affective states can be continuously monitored *via* these signals. Furthermore, signal features extracted from specific brain regions have been demonstrated to correlate with emotion dynamics (Alarcao and Fonseca, 2019).

Since EEG not only reflects emotional states but also indicates other cognitive activities in the brain, it is necessary to choose independent variables to discriminate emotions from EEG rhythms and lobe locations. Researchers have observed a correlation between emotional activity and EEG signals. Features that can reflect emotional changes are mainly found in the right occipital lobe and parietal lobe for the alpha band, the parietal lobe and temporal lobe for the beta band, and the left frontal lobe and right temporal lobe for the gamma band (Davidson et al., 1985; Ray and Cole, 1995; Schutter et al., 2001; Guntekin and Basar, 2007).

Electroencephalography measurements vary with the level of cognitive stimulation (Klimesch, 1999; Anderson and Bratman, 2008). This makes EEG an appropriate technology for measuring the cognitive load in educational psychology. The conventional analysis of the alpha, beta, theta, delta, and gamma bands has been reported as the foundation for several EEG-based models of mental load (Klimesch, 1999; Kirmizi-Alsan et al., 2006; Khosrowabadi et al., 2010). Theta and alpha oscillations are more sensitive to task difficulty. Several researchers have proven that alpha and theta activities are related to task difficulty or cognitive load for various demanding tasks. Beta waves have been proven to be related to perception and cognition (Rangaswamy et al., 2002). In summary, the theta, alpha, and beta bands are most related to cognitive load.

It has already been proven that the use of different learning design features impacts learners’ emotions and cognitive load (Um et al., 2012; Mayer and Estrella, 2014; Plass et al., 2014, 2020; Park et al., 2015). In our previous study (Liu et al., 2021), we tested the effectiveness of color coding on the learning of computer programming students who were learning from video lectures. The results showed that a color-coded design is more beneficial than a grayscale design, as indicated by lower EEG cognitive load and better learning performance.

In the present study, we aimed to investigate the relationship between emotional activity and cognitive load in multimedia learning from an emotion dynamics perspective, using EEG signals. We aimed to investigate the trajectory of learners’ emotional changes under different cognitive load states and explore the modulatory effect of cognitive load on emotional activity. Specifically, we propose the following hypothesis: Learners in higher cognitive load states undergo slower emotional changes than those in lower cognitive load states. That is, emotional activity is negatively associated with cognitive load. To the best of our knowledge, this issue has not been studied by the neuroscientific method from an emotion dynamics perspective.

## Materials and Methods

### Participants

The participants were 42 graduate students (31 males vs. 11 females) recruited from the Zhejiang University of Science and Technology (ZUST). All the participants were over 18 years old (*M* = 20.81, SD = 1.13). The study was approved by the ethics committee of the School of Information and Electronic Engineering, ZUST. All participants had taken C/C++ courses to ensure that they had the necessary programming foundation. Prior knowledge was assessed to ensure that participants had the same level of prior knowledge (Liu et al., 2021). All participants signed an informed consent form before the experiment and received a small gift at the end of the study to thank them for their time and effort.

### Tasks

**FIGURE 1:**
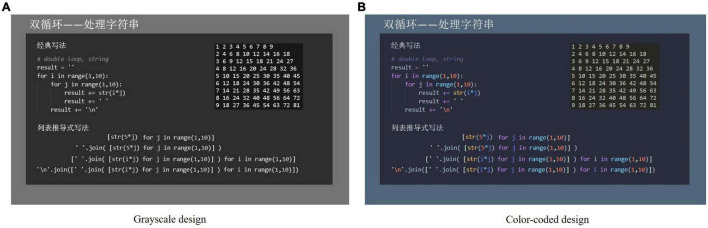
Two video lecture conditions **(A)** Grayscale design; **(B)** color-coded design.

In this study, we used the same materials as in our previous study (Liu et al., 2021; Figure 1). One video lecture was grayscale (Figure 1A), with a black background and white text. The other video lecture was color-coded (Figure 1B). We used the “Palenight Theme” for code highlighting as it is widely used in computer programming. Each video lecture included the same PowerPoint slideshow accompanied by a lecture given by a professor who frequently taught this topic. The video lectures were identical in terms of speed, sound, and light. The materials were not self-paced, and did not allow learners to start, stop, or replay short sequences. Each video lecture lasted approximately 5 min and introduced the topic of “List Expression in Python” in Chinese.

The participants were randomly assigned to one of the following two video lecture conditions: a grayscale group and a color-coded group. As a result, one group received learning material with a grayscale design. The other group received learning material with a color-coded design.

### Procedure

Before starting the experiment, all participants washed their hair to lower impedance. Each participant then learned about the experimental procedure and signed a consent form. After that, the participants closed their eyes for 3 min so that we could take a baseline measurement of their EEG signal features. Next, participants viewed one of the video lectures and then completed a cognitive load questionnaire immediately after viewing the lecture (Liu et al., 2021). Participants’ EEG signals were assessed while they watched the videos. The experiment was conducted in a laboratory which free of electromagnetic interference for approximately 20 min.

### Data Collection and Analysis

**FIGURE 2:**
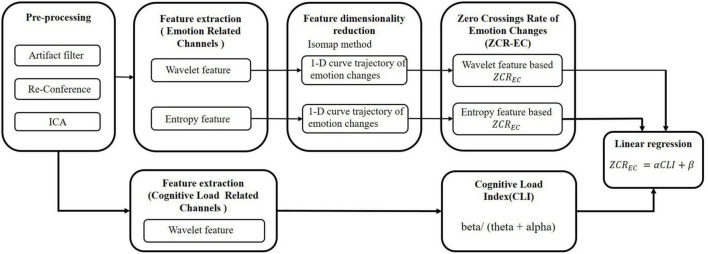
Flowchart of the proposed method.

Figure 2 shows the flowchart of the proposed method. First, we preprocess the EEG signals. To represent emotion changes, we extracted emotion-related features and put those features into a dimensionality reduction model to obtain a one-dimensional trajectory of emotional changes. Then, we quantitatively characterized the emotional changes. Meanwhile, we extracted cognitive load related features to analyze the degree of cognitive load. Lastly, we designed a linear regression model to fit the relationship between emotional changes and cognitive load for multimedia learning.

#### Electroencephalography Data Collection

Electroencephalography was recorded at 15 electrode sites (Fp1, Fp2, F7, F3, Fz, F4, F8, T7, Cz, T8, P7, Pz, P8, O1, and O2) positioned according to the international 10/20 system (Jasper, 1958). CPz served as a reference during recording. The data were referenced to the average of all electrodes. The ground electrode was located at AFz. EEG data were recorded (OpenBCI) at a 125 Hz sampling rate (OpenBCI, Inc.) using active electrodes.

#### Data Preprocessing

The EEG data preprocessing procedure was composed of the following steps:

(1) The power-frequency interference was eliminated by band-stop filter.
(2) The DC components were removed at 1 Hz using a finite impulse response (FIR) high-pass filter. We then removed other artifact noises at high frequencies using a FIR low-pass filter at 50 Hz.
(3) We referenced the EEG data by subtracting the average of all collected electrodes from each individual electrode.
(4) We conducted independent component analysis to remove EOG and eye artifacts (Jung et al., 2000; Delorme et al., 2007).
(5) The Hilbert Huang Transform (HHT) (Huang et al., 1998) technique was used to calculate the power spectrum of each EEG epoch (4 s) with a frequency resolution of 0.1 Hz.
(6) We computed the power of the delta (0–3.9 Hz), theta (3.9–7.8 Hz), alpha (7.8–13.7 Hz), beta (13.7–29.3 Hz), and gamma (29.3–46.9 Hz) rhythms by averaging the spectral powers in the corresponding frequency bands. Consequently, the number of EEG features for each participant was 5 (no. EEG rhythm features) × 15 (no. of channels) = 75.

### Feature Extraction

#### Power Spectrum Feature: Hilbert Huang Transform

Spectral analysis is a useful method for revealing the statistical characteristics of stochastic data in neuroscience research. Fourier spectral analysis is a powerful tool for examining global energy-frequency distributions, but it has some crucial restrictions, such as the system must be linear and the data must be strictly stationary. However, most natural physical processes are non-linear and non-stationary. The HHT (Huang et al., 1998) provides a full informational representation of non-linear and non-stationary data, especially for time-frequency representations. It can discriminate between the natural amplitude and frequency modulations that often occur in non-linear systems. The basic idea of HHT is to combine empirical mode decomposition (EMD) and the Hilbert transform. The HHT can preserve the characteristics of various frequencies. This is an important advantage of HHT because real-world signals usually occur at multiple and different time intervals.

The HHT procedure consists of the following steps:

(1) EMD decomposes the data into a finite number of intrinsic mode function (IMF) components and applies the Hilbert spectral analysis (HSA) method to the IMFs to obtain instantaneous frequency data.

(2) The Hilbert transform was applied to each component, and the instantaneous frequency was calculated according to the equation. HSA is a method that checks the instantaneous frequency of each IMF as a function of time. The final result is a frequency-time distribution of the signal amplitude (or energy) to be designated as the Hilbert spectrum, which allows the identification of local features.

(3) After performing the Hilbert transform on each IMF component *a_j_*, the data can be expressed in the following form:


(1) $$X\,\left(t\right)=r\,e\,a\,l\,\sum_{j=1}^{n}a_{j}\,\left(t\right)\,e^{i\,\int \omega_{j}\,\left(t\right)\,dt}$$

In this study, we used the HHT method to build the frequency-time distribution of signal energy, which permits the identification of local features and physical meanings.

#### Frequency Domain Feature: Wavelet Feature

Wavelet transform is a typical method for time-frequency analysis which maintains the idea of short-time Fourier transform local signal processing and overcomes the shortcoming of an unalterable window size, making it an ideal tool for signal time-frequency analysis and processing. The wavelet feature is the general time–frequency domain feature used for EEG signal analysis (Rosso et al., 2001).

In this study, we also found that Daubechies 10 was the most suitable for accurate analysis of EEG signals (Ganorkar et al., 2019). The EEG signal was decomposed into 32 components using sixth-order Daubechies wavelet package decomposition. We decomposed these components to compute classical EEG rhythms, as shown in Table 1.

**TABLE 1:** EEG rhythms.

EEG rhythm	Components	Frequency range (Hz)
Delta	1–2	0–3.9
Theta	3–4	3.9–7.8
Alpha	5–7	7.8–13.7
Beta	8–15	13.7–29.3
Gamma	15–24	29.3–46.9

To find general features we used the wavelet energy ratio *R_i_*to represent the i-th EEG rhythm energy *E_j_*.


(2) $$R_{i}=\frac{E_{j}}{E_{t\,o\,t\,a\,l}}$$

Then, the wavelet entropy can be defined as:


(3) $$W_{e}=-\sum_{i=1}^{n}R_{i}\,\ln R_{i}$$

#### Non-linear Dynamical Feature: Entropy Feature

Electroencephalography data are highly complex and non-linear. In recent years, many researchers have proposed methods for analyzing EEG signals using non-linear dynamics theory. Among these, approximate entropy, sample entropy, permutation entropy, and state space correlation entropy are important tools used to quantify the complexity of a time series. They can keep the information captured in an original time series as “whole” as possible, thus proving to be more effective than other methods in this respect.

By using sliding windows, raw signal can be divided into several pieces. Then the distance matrix D between each two pieces can be calculated. We employed two entropy algorithms for complexity estimate.

As proposed by Pincus (1991), approximate entropy can be used to consider the complexity and integrated degree of a sequence. It uses a non-negative number to represent the complexity and irregularity of the time series and can reflect the occurrence rate of new sequence information in the time series (Wan et al., 2021). If the time series is more irregular and complex, then the corresponding approximate entropy is larger.

Consider a signal with n sample points. First, we set a distance threshold F, then calculated the proportion $C_{i}^{m}\,\left(t\right)$ of elements greater than F in each row. Here m is the windows length, i is the row index. Logarithmic mean can then be calculated:


(4) $$\Phi^{m}\,\left(t\right)=\frac{1}{n-m+1}\,\sum_{i=1}^{n-m+1}l\,n\,C_{t}^{m}\,\left(t\right)$$

By change windows length we can give the approximate entropy expression:


(5) $$\mathrm{ApEn}\,\left(t\right)=\Phi^{m}\,\left(t\right)-\Phi^{m+1}\,\left(t\right)$$

Sample entropy (Richman and Randall, 2000) has the following advantages: (1) the calculation of sample entropy does not rely on the duration of the data, and (2) sample entropy displays greater consistency. Similar to the approximate entropy, the greater the sample entropy, the lower the sequence’s self-similarity and the more complex the sample sequence.

The calculation of sample entropy is slightly different. We do not calculate this window when calculate logarithmic mean.


(6) $$\Phi^{m}\,\left(t\right)=\frac{1}{n-m}\,\sum_{i=1}^{n-m}C_{t}^{m}\,\left(t\right)$$

The sample entropy can be defined as:


(7) $$\mathrm{SampEn}\,\left(t\right)=l\,n\,\Phi^{m}\,\left(t\right)-l\,n\,\Phi^{m+1}\,\left(t\right)$$

Permutation entropy (Bandt and Pompe, 2002) is a kind of quantification measure index of complexity. In a dynamic system, permutation entropy can capture the order relationship between time series values, and extract the probability distribution of the ordinal pattern. The first step is to partition the time series into same length pieces. Set the length is *D*. Between pieces there is a series overlapping part. The second step is to stack those pieces into a matrix *Y*, which is also called state matrix. The final step is to sum all entropy of the full permutation in the *D*-wise as:


(8) $$P\,E\,n\,\left(D\right)=-\sum_{i=1}^{D!}p_{i}\,{\text{log}}_{2}\,p_{i}$$

The state space correlation entropy is an extension of permutation entropy (Tripathy et al., 2017). After obtain state matrix, we calculate the auto correlation matrix by matrix multiplication *Y*^T^*Y*. Then we use the upper triangular elements to calculate the histogram and probability *p*_*k*_ of the k-th bin. Finally, the state space correlation entropy can be defined as:


(9) $$S\,S\,C\,E\,n\,\left(K\right)=-\sum_{i=1}^{K}p_{i}\,{\text{log}}_{2}\,p_{i}$$

### Feature Dimensionality Reduction: Isomap Method

Emotions usually fluctuate across time. Wang et al. (2012) employed a CFS method to find subject-independent features of EEG signals which related to emotion trajectory. In this step, we chose Fp1, F7, T7, Cz, T8, P8, and O2 as research lobes which were consistent with the findings reported by Wang. These lobes were mostly related with emotion activity, allowing us to calculate the emotion trajectory curve using the Isomap method.

First, with the help of the CFS method, we chose the top-15 subject-independent features, following Wang et al. (2014). Those features were then put into the Isomap model to reduce them into a one-dimensional curve representing an emotion trajectory curve.

An Isomap attempts to maintain the internal geometry of the non-linear data by using the geodesic manifold distances between data points. The algorithm can be divided into three steps (Tenenbaum et al., 2000).

(1) Construct neighborhood graph. For each sample point, we first search the k nearest neighbors. In the original spaces, we construct a neighborhood connecting graph by calculating the distance of each pair of points as edge weights.

(2) Compute shortest paths. The *d*_*ij*_ means the paths of each pair of points i, j. Then the shortest paths can be computed using the Dijkstra method with the neighborhood graph.

(3) Construct d-dimensional embedding. With the shortest paths, we can represent the data as a matrix $D=\left\{d_{i\,j}^{2}\right\}$, expressing the geodesic distance of point pairs. Classical multidimensional scaling (MDS) can then be applied to this matrix to construct an embedding of the data that best maintains the manifold’s estimated internal geometry.

(4) Input the selected features into the Isomap model and reduce to one dimension. This one-dimension curve is the trajectory of emotion changes (Wang et al., 2014).

### Zero Crossings Rate of Emotional Changes

Petrantonakis and Leontios (2010) used a zero-crossing count to express the EEG oscillations. A higher zero-crossing rate (ZCR) indicates a more violent EEG oscillation. This can provide information about the user’s emotional status, such as time-dependent emotional trends. Because the Isomap method output curve has zero mean, we followed Petrantonakis’s conclusion and used ZCR as the quantitative characterization of emotional changes (*ZCR*_*EC*_).

The EEG time series *s*(*n*) related to emotional changes is converted into a zero-mean series *Z*(*n*),*n* = 1,2,,*N*. Apply *M* high-pass filters are applied to series *Z*(*n*), and *k* represents the order of the filters, *k* = 1,2,,*M*.


(10) $$L_{k}\,\left\{Z\,\left(n\right)\right\}=\sum_{j=1}^{k}\frac{\left(k-1\right)!}{\left(j-1\right)!\,\left(k-j\right)!}\,{\left(-1\right)}^{j-1}\,Z\,\left(n-j+1\right)$$

Based on *L*_*k*_{*Z*(*n*)} to construct binary time series *X*_*n*_(*k*),


(11) $$X_{n}\left(k\right)=\left\{ \begin{array}{c} 1,L_{k}\,\left\{Z\,\left(n\right)\right\}\geq 0\\ 0,L_{k}\,\left\{Z\,\left(n\right)\right\}<0 \end{array},k=1,2,,M;n=1,2,,N\right.$$

The desired simple*ZCR*_*EC*_are then estimated by counting symbol changes in *X*_*n*_(*k*).


(12) $$Z\,C\,R_{E\,C}=\sum_{n=2}^{N}{\left[X_{n}\,\left(k\right)-X_{n-1}\,\left(k\right)\right]}^{2}$$

### Cognitive Load Index

According to Pope et al. (1995), task engagement can be reflected by beta/ (theta + alpha). There is a direct correlation between the EEG engagement index and task load (Klimesch, 1999), and this index is very effective in quantifying the state of an individual’s mental load.

In our previous research (Liu et al., 2021), we demonstrated that one group that received learning material with a grayscale design had a higher cognitive load than another group that received learning material with a color-coded design. We calculated the main effect of different electrodes on the delta, theta, alpha, beta, and gamma powers under both conditions. The analysis results indicated that the Fp1, Fp2, F3, T8, Cz, P8, O1, and O2 lobes for alpha power, the Fp1, F3, F7, F8, Fz, F4, T8, and Cz lobes for beta power, and all lobes for theta power showed significant differences. In this study, we sampled these cognitive load related EEG signals to analyze the degree of cognitive load, which we defined as cognitive load index (CLI).

### Linear Regression Model

Using the least square method, we constructed a linear regression model to fit the relationship between emotional changes and cognitive load in multimedia learning.


(13) $$Z\,C\,R_{E\,C}=\alpha \,C\,L\,I+\beta$$

Larger α values reflect stronger *ZCR*_*EC*_ modulation by CLI.

## Results

### Electroencephalography Frequency-Domain Features

Data from four participants were deleted because of low signal quality. As a result, the grayscale and color-coded design groups included data from 19 participants each (*n* = 38).

The Shapiro–Wilk test was used to test the normality of the EEG power features (*p* > 0.05). One-way ANOVA was conducted with the between-subject factors of the two design conditions and delta, theta, alpha, beta, and gamma power as dependent variables. One-way ANOVA revealed a significant difference between the experimental conditions in theta, alpha, beta, and gamma power [F (1, 37) = 9.36, *p* = 0.004, $\eta_{p}^{2}$ = 0.206; F (1, 37) = 4.43, *p* = 0.042, $\eta_{p}^{2}$ = 0.110; F (1, 37) = 4.89, *p* = 0.034, $\eta_{p}^{2}$ = 0.119; F (1, 37) = 4.40, *p* = 0.043, $\eta_{p}^{2}$ = 0.109]. There was no difference in delta power [F (1, 37) = 0.233, *p* = 0.632, $\eta_{p}^{2}$ = 0.006].

**FIGURE 3:**
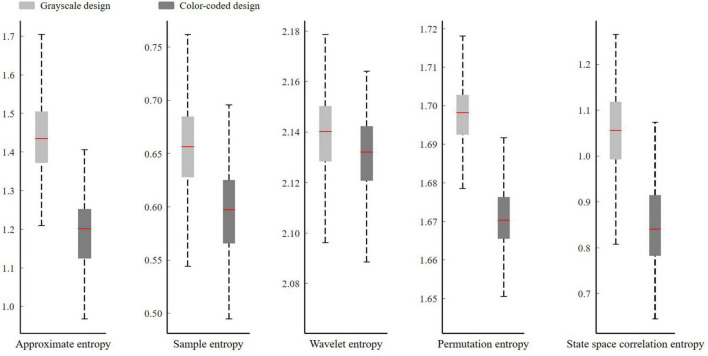
Boxplot of approximate entropy, sample entropy, permutation entropy, state space correlation entropy, and wavelet entropy in the two conditions.

Figure 3 shows the electrode frequency distribution maps for the grayscale and color-coded designs. The HHT was applied to each EEG channel of all the samples obtained above, and the transformed results were normalized to produce input data suitable for correlation-based feature selection. As illustrated in Figure 2, we found obvious differences between the theta, alpha, beta, and gamma bands.

### Entropy Features

In this study, we studied four different entropy values of EEG time-domain signals (approximate entropy, sample entropy, permutation entropy, and state space correlation entropy) and compared them with EEG frequency domain signals (wavelet entropy).

**FIGURE 4:**
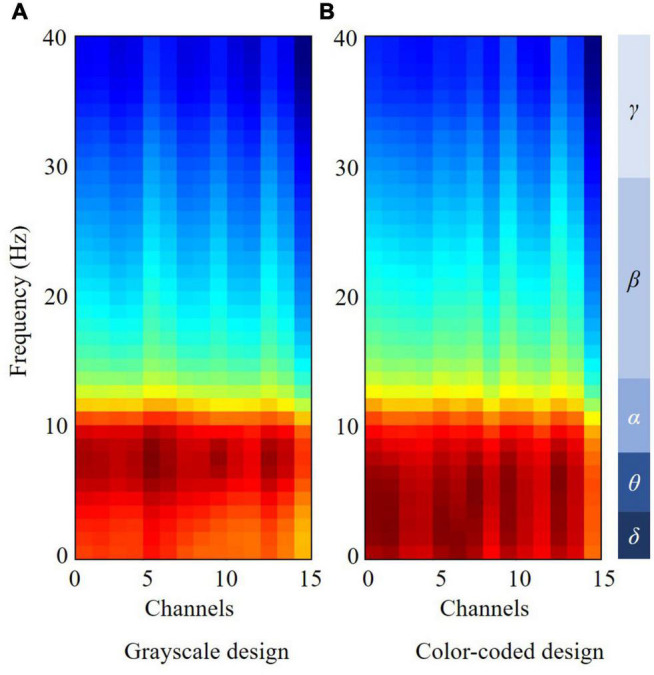
Electrode-frequency distribution maps for grayscale design and color-coded design.

The Shapiro–Wilk test was used to test the normality of the entropy features (*p* > 0.05). Figure 4 shows the boxplot of approximate entropy, sample entropy, permutation entropy, state space correlation entropy, and wavelet entropy in the two conditions. One-way ANOVA revealed statistically significant differences across the experimental conditions on approximate entropy [F (1, 37) = 624.03, *p* < 0.001, η${}_{p}^{2}=$ 0.617], sample entropy [F (1, 37) = 191.58, *p* < 0.001, η${}_{p}^{2}$ = 0.331], permutation entropy [F (1, 37) = 1,037.46, *p* < 0.001, η${}_{p}^{2}=$ 0.727], state space correlation entropy[F (1, 37) = 463.03, *p* < 0.001, η${}_{p}^{2}=$ 0.543] and wavelet entropy [F (1, 37) = 27.31, *p* < 0.001, η${}_{p}^{2}=$ 0.069].

**FIGURE 5:**
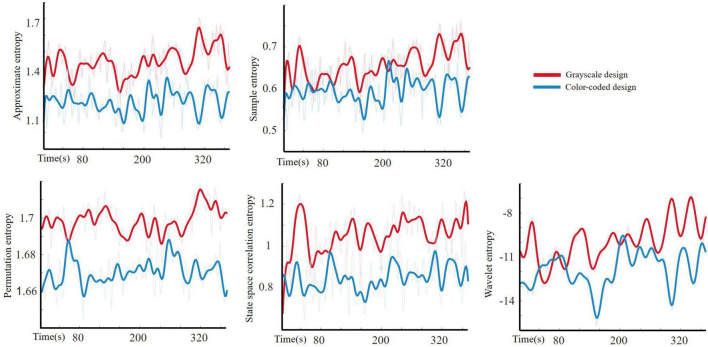
The average approximate entropy, sample entropy, permutation entropy, state space correlation entropy, and wavelet entropy of all participants engaging with grayscale design and color-coded design.

The average approximate entropy, sample entropy, permutation entropy, state space correlation entropy, and wavelet entropy of all the participants with different video lecture designs are shown in Figure 5. The study revealed that the participants who watched the grayscale material had higher approximate entropy, sample entropy, permutation entropy, state space correlation entropy, and wavelet entropy than those who watched the color-coded design material. As shown in Figure 5, the approximate entropy, permutation entropy, and state space correlation entropy more clearly distinguished between the two types of video lecture designs, while the distinction between the two lecture designs was less obvious for sample entropy and wavelet entropy. These were consistent with one-way ANOVA results that approximate entropy, permutation entropy, and state space correlation entropy had large effect size, the sample entropy had medium effect size, and the wavelet entropy had small effect size.

### Cognitive Load Index

**FIGURE 6:**
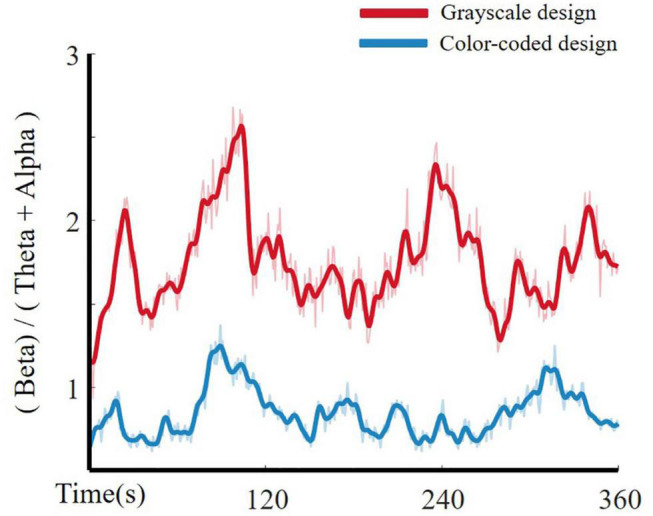
Cognitive load curves for grayscale design and color-coded design.

The cognitive load curve based on Pope’s index beta/(theta + alpha) for cognitive load related channels is illustrated in Figure 6.

As shown in Figure 6, the beta/(theta + alpha) index is suitable for distinguishing higher cognitive load from lower cognitive load in multimedia learning. Participants had significantly higher beta/(theta + alpha) power when learning through color-coded video lectures than through grayscale lectures.

**FIGURE 7:**
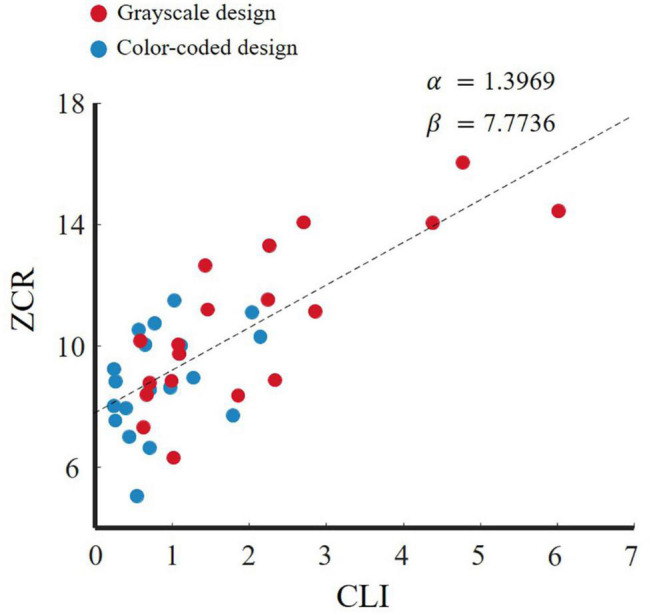
Fitting curve of CLI and ZCR of EEG time-domain raw signals.

The ZCR can also be used to directly observe the intensity of EEG signal changes. By choosing the same channels as those described in section “Feature Dimensionality Reduction: Isomap Method,” we represent the ZCR -CLI fitting curve in Figure 7. The modulation parameter α was fitted to 1.3969 which indicated that a larger CLI caused more violent fluctuations in the EEG time-domain raw signals. With entropy as a complexity measure (Parbat and Chakraborty, 2021), more violent fluctuations in the EEG time-domain may induce higher entropy. This provides evidence that the higher the entropy, the higher the cognitive load in cognitive processing.

### Linear Regression

As illustrated in Figure 2, we employed a linear regression fitting method to study the relationship between *ZCR*_*EC*_ and CLI. We conducted this study from two perspectives. One is the frequency domain method (wavelet feature), and the other is the non-linear dynamic method (entropy feature).

#### Frequency Domain Method

**FIGURE 8:**
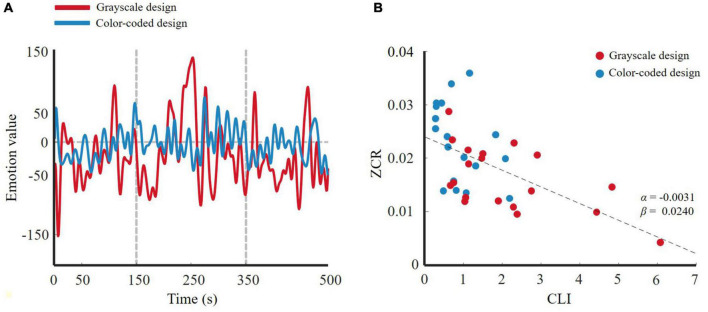
**(A)** 1-D trajectory of emotion change of wavelet feature for two designs; **(B)** *ZCR*_*EC*_-CLI fitting curve of wavelet feature.

For the two participant groups, after feature dimension reduction, the emotion trajectory curves are shown in Figure 8A. The red line represents the participants who were given the color-coded material and the blue line represents those who were given the grayscale material. The participants had more obvious changes in their emotional trajectory when learning through color-coded video lectures than through grayscale lectures.

For the linear regression model in Equation 13, the *ZCR*_*EC*_-CLI fitting curve for all subjects is shown in Figure 8B. The modulation parameter α = −0.0031 indicates that a larger CLI leads to slower emotional change. Stated differently, the participants who received the color-coded learning material experienced lower cognitive load, and their emotional trajectory changed more obviously. That is, emotion changes more actively in a lower cognitive load state than in a higher cognitive load state. This result was consistent with our hypothesis.

#### Non-linear Dynamic Method

**FIGURE 9:**
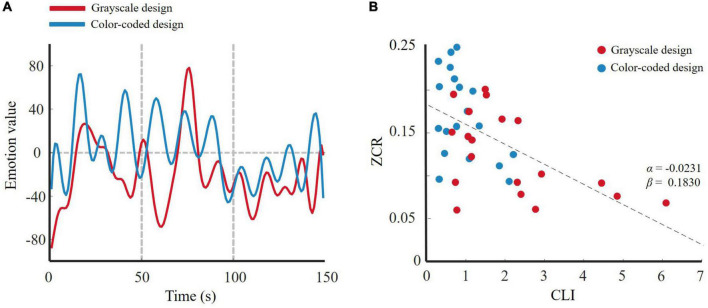
**(A)** 1-D trajectory of emotion change of approximate entropy for two design; **(B)** *ZCR*_*EC*_-CLI fitting curve of approximate entropy.

**FIGURE 10:**
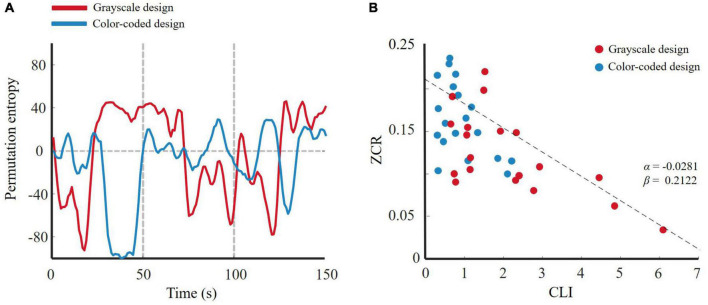
**(A)** 1-D trajectory of emotion change of permutation entropy for two designs; **(B)** *ZCR*_*EC*_-CLI fitting curve of permutation entropy.

**FIGURE 11:**
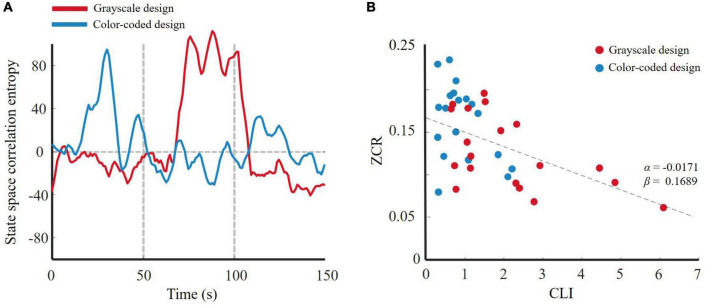
**(A)** 1-D trajectory of emotion change of state space correlation entropy for two designs; **(B)** *ZCR*_*EC*_-CLI fitting curve of state space correlation entropy.

Vacha-Haase and Thompson (2004) reported no matter which statistic method was used that an effect size 0.5 meant something very different. One-way ANOVA revealed that the effect sizes of approximate entropy, permutation entropy, and state space correlation entropy were larger than 0.5. Therefore, we chose them as the entropy features to fit the linear regression model. Approximate entropy, permutation entropy, and state space correlation entropy can also be applied *via* a dimensionality reduction method such as Isomap. In particular, when calculating the entropy, we set the window width to 5 s, approaching a cycle of emotional changes. A typical result is shown in Figures 9A, 10A, 11A. The participants who received the color-coded video lectures, that is, those in a lower cognitive load state, experienced more active emotional changes. Figures 9B, 10B, 11B show the *ZCR*_*EC*_-CLI fitting curve for all participants. Obviously, a higher cognitive load caused a slower change in the entropy one-dimensional reduced curve. In other words, emotional activity is negatively associated with cognitive load in multimedia learning. This result supports our hypothesis.

## Discussion

The main goal of this study was to investigate the relationship between emotional activity and cognitive load in multimedia learning. We examined this issue from an emotion dynamics perspective using EEG signals. A computer programming learning experiment was conducted. We investigated the learners’ trajectory of emotional changes under different cognitive load states and explored the influence of higher and lower cognitive loads on emotional activity. We conducted this study from two perspectives. One is the frequency domain method (wavelet feature), and the other is the non-linear dynamic method (entropy features). The results indicated that participants in lower cognitive load states had more active changes in their emotional trajectory than those in higher cognitive load states. This observation is consistent with the findings of several emotion dynamics studies (Kuppens et al., 2010; Kuppens and Verduyn, 2017), confirming that emotional activity is negatively associated with cognitive load.

Our results are in line with the emotional design hypothesis (e.g., Mayer and Estrella, 2014; Plass et al., 2014). The emotional design hypothesis suggests that learning material with color effects is a positive emotional design, while grayscale material has a neutral emotional design. Positive emotion design can reduce cognitive load (Um et al., 2012; Plass et al., 2014; Park et al., 2015). Figure 4 illustrates that the participants who watched the color-coded learning material had lower entropy than those who watched the grayscale learning material. Figure 7 shows that the lower the entropy, the lower the cognitive load. These results demonstrated that the participants who received the color-coded learning material had a lower cognitive load. The results provide empirical evidence for the emotional design hypothesis.

We selected EEG features with significant differences between the two groups to test our hypothesis from two perspectives. We used wavelet features to perform the frequency domain method analysis and entropy to conduct a non-linear dynamic method analysis. The results of these analyses were consistent with our hypothesis. Figures 8A–11A demonstrate that the participants in the grayscale design group underwent slower emotional changes than those in the color-coded design group. Because the color-coded design group was associated with lower cognitive load and the grayscale design group was associated with higher cognitive load, based on the linear regression model that we promoted, emotional activity was negatively associated with cognitive load both in the color-coded and grayscale design group (see Figures 8B–11B). Neuroscience research has shown that the late positive potential (LPP) of EEG signals is significantly related to emotional stimuli (Weinberg and Hajcak, 2010). Evidence has shown that compared to the low-load task, the high-load task decreased the LPP amplitude (MacNamara et al., 2011; Gan et al., 2017). Cognitive load reduces activity in emotion response brain regions (Sun et al., 2020). Our results are consistent with prior neuroscience research using EEG signals.

This study provides further explanation for our previous research (Liu et al., 2021). The color-coded design as a positive emotion design increased germane cognitive load and learning motivation (Um et al., 2012). In addition, learning is compromised when an individual’s emotional diversity and emotional experience intensity are below optimal levels (Pekrun, 2006; Pekrun and Stephens, 2010). Learners in a lower cognitive load state can promote their emotional activity to benefit from learning.

ICALM assumes that every information-processing step in the learning process is emotional and cognitive. According to Baddeley and Hitch (1994), working memory is a resource-limited system, and the simultaneous performance of the two tasks introduces a competition for cognitive resources. Our study demonstrated that EEG emotional activity is negatively associated with cognitive load in multimedia learning, providing neuroscientific evidence for ICALM theory.

This study has some limitations that should be addressed in future studies. First, to create standardized learning conditions, the study was conducted in a laboratory setting. Future studies should be conducted in realistic learning settings in order to confirm their ecological validity. Second, we did not strictly control for metacognitive factors. ICALM recognizes that metacognition mediates the cognitive processing of multimedia information. Although we controlled for participants’ prior knowledge, metacognition also includes understanding and controlling for other cognitive processes (Lindnera et al., 2021). Future research should test participants’ metacognitive capacity, which might influence their emotion and cognitive processing in multimedia learning. Third, the number of participants were relatively small, and the research had not considered the gender factor. Gender differences are an important topic in computer thinking research. Gender differences in self-efficacy and interest may affect programming learning (Allaire-Duquette et al., 2022), these may have influence on learners’ emotion and cognitive load during the experiment. Future studies should take gender difference as the experimental control variable for further research. Moreover, future researches should use different materials to assess the generalizability of the current findings.

## Conclusion

The present study explores multimedia learning, a topic that has received much interest and is of considerable importance at present because of the COVID-19 pandemic and increased online learning. Based on the research method of emotional dynamics, the present study discusses the relationship between cognitive load and emotional activity in multimedia learning and provides EEG neuroscience evidence that emotional activity is negatively associated with cognitive load. To our knowledge, this is the first study to fit cognitive load effects on emotional activity from a neural perspective. This study provides neural evidence for the emotional design hypothesis and ICALM theory. This research also provides empirical support for the argument that learning material should reduce learners’ cognitive load to keep learner’s emotional experience at optimal levels to benefit learning.

## Data Availability Statement

The datasets presented in this study can be found in online repositories. The names of the repository/repositories and accession number(s) can be found below: https://github.com/zeron21/Multimodal_Data.git.

## Ethics Statement

The studies involving human participants were reviewed and approved by the Ethics Committee of the School of Information and Electronic Engineering, Zhejiang University of Science and Technology. The participants provided their written informed consent to participate in this study.

## Author Contributions

All authors listed have made a substantial, direct, and intellectual contribution to the work, and approved it for publication.

## Conflict of Interest

The authors declare that the research was conducted in the absence of any commercial or financial relationships that could be construed as a potential conflict of interest.

## Publisher’s Note

All claims expressed in this article are solely those of the authors and do not necessarily represent those of their affiliated organizations, or those of the publisher, the editors and the reviewers. Any product that may be evaluated in this article, or claim that may be made by its manufacturer, is not guaranteed or endorsed by the publisher.
